# Cost-Effectiveness Analysis of Stereotactic Ablative Body Radiotherapy for the Treatment of Oligometastatic Tumors versus Standard of Care

**DOI:** 10.3390/curroncol28030172

**Published:** 2021-05-13

**Authors:** Adam J. N. Raymakers, David Cameron, Scott Tyldesley, Dean A. Regier

**Affiliations:** 1 Cancer Control Research, BC Cancer, Vancouver, BC V5Z 1L3, Canada; araymakers@bccrc.ca (A.J.N.R.); david.cameron@cadth.ca (D.C.); 2 Faculty of Health Sciences, Simon Fraser University, Burnaby, BC V5A 1S6, Canada; 3 Radiation Therapy Program, BC Cancer, Vancouver, BC V5Z 4E6, Canada; styldesl@bccancer.bc.ca; 4 Faculty of Medicine, University of British Columbia, Vancouver, BC V6T 1Z3, Canada; 5 School of Population and Public Health, University of British Columbia, Vancouver, BC V6T 1Z3, Canada

**Keywords:** cost-effectiveness analysis, cost-utility analysis, economic evaluation, radiotherapy, SABR, health technology assessment, early health technology assessment, economic evaluation

## Abstract

Background: Recent clinical trial results reported that stereotactic radiotherapy (SABR) may improve survival for patients with oligometastatic (OM) cancer. Given that these results come from a phase II trial, there remains considerable uncertainty about this finding, and about the cost-effectiveness of SABR for patients with OM cancer. In this analysis, we estimate the cost-effectiveness of SABR for oligometastatic cancer patients. Methods: A probabilistic time-dependent Markov model was constructed to simulate treatment of oligometastatic cancer patients over five- and ten-year time horizons. The primary data source was the phase II, Stereotactic Ablative Radiotherapy for the Comprehensive Treatment of Oligometastases (SABR-COMET )trial and supplemented with data from the literature. We estimated the effect of SABR and the standard of care (SoC) using quality-adjusted life-years (QALYs). Costs were measured from a provincial payer perspective (2018 Canadian dollars). Results: In the reference case analysis (five-year time horizon), SABR was associated with additional incremental costs of CAD 38,487 and an incremental QALY gain of 0.84. This resulted in an incremental cost-effectiveness ratio (ICER) of CAD 45,726 per QALY gained. Over a ten-year time horizon, the increased uncertainty in the long-term effectiveness of SABR resulted in an ICER of CAD 291,544 per QALY gained. Estimates from the probabilistic analysis indicated that at a willingness-to-pay (WTP) threshold of CAD 50,000 and CAD 100,000 per QALY gained, there is 54% and 78% probability (respectively) that SABR would be cost-effective using the five-year time horizon. Conclusions: The adoption of SABR therapy requires a considerable upfront capital investment. Our results suggest that the cost-effectiveness of SABR is contingent on the uncertainty in the evidence base. Further clinical trials to confirm the effectiveness of SABR and research into the real-world costs associated with this treatment could reduce the uncertainty around implementation of the technology.

## 1. Introduction

Stereotactic ablative body radiotherapy (SABR) is a an external beam radiotherapy (RT) technique used to deliver high doses of radiation with high conformality to malignant lesions in the body [1,2]. Treatment with SABR is designed to provide a high dose of ionizing radiation to extra-cranial tumors while minimizing the radiation exposure to the surrounding tissue, using either single-dose (fractions) or a small number of fractions [3]. Treatment with SABR provides precision at a higher dose per fraction, and higher conformality compared to conventional radiotherapy and uses advanced imaging guidance techniques to target tumors with millimeter-scale accuracy while sparing neighboring healthy tissue from intense radiation. This permits higher doses of radiation to be administered than with previous RT techniques with fewer fractions, and allows for a high target dose with a steep dose gradient beyond the target [3]. This level of precision with SABR is particularly valuable when tumors are proximal to other organs or critical structures. Treatment with SABR can be used for a variety of histologies, anatomic sites, and stages of cancer [2]. Moreover, SABR can be used as a first-line treatment option, for example, for non-small cell lung cancers (NSCLC) [4] where it is often used when surgery is not an option for patients who are inoperable due to age or comorbidity. SABR can also be used in the metastatic setting to alter disease progression and survival. In this setting SABR is usually reserved for patients with a small number of metastases (up to 5), referred to as oligometastatic disease [5,6].

The international, multi-center, phase II, Stereotactic Ablative Radiotherapy for the Comprehensive Treatment of Oligometastases (SABR-COMET) trial compared palliative care to SABR plus palliative care for patients with oligometastases (1–5 metastatic lesions) [7]. Trial results suggested that treatment with SABR was associated with an increase in median overall survival (OS) of 13 months (hazard ratio (HR): 0.57; 95% CI: 0.30–1.10) compared to those receiving palliative care, and patients’ progression-free survival (PFS) time doubled [7,8].

The objective of this analysis, given recently published evidence supporting the use of SABR, is to estimate the cost-effectiveness of SABR for treatment of patients with oligometastatic disease compared to the standard of care from the British Columbian provincial payer perspective. 

## 2. Methods

### 2.1. Patient Population

The patient population for this analysis is assumed to reflect that of the SABR-COMET trial [8]. The multi-center trial examined patients aged 18 or older with a controlled primary tumor and one to five metastatic lesions, Eastern Cooperative Oncology Group (ECOG) score of 0–1, and a life-expectancy of at least six months. Approximately 77% of all SABR-COMET patients had primary tumors of breast, colorectal, lung, or prostate cancer, with the remainder of tumor sites unreported (Tables S2–S5). Evidence from this trial informs the assumption that patients with a limited number of metastases may have improved survival if all satellite tumors are treated [7]. Our model deviates from SABR-COMET in that we evaluate the cost-effectiveness of SABR for oligometastatic disease in comparison to systemic therapy (as the SoC), as opposed to palliative care, to better reflect real-world practice.

### 2.2. Modeling Approach

**Figure 1:**
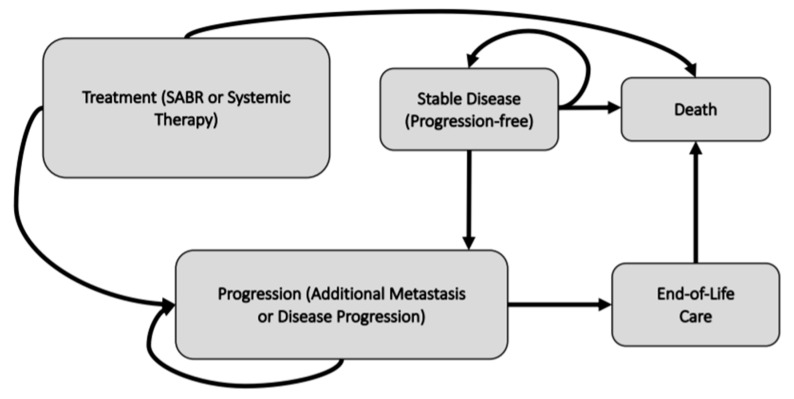
Conceptual diagram representing the SABR treatment arm and comparator arm (systemic therapy) of the economic model for patients with oligometastatic disease. The model arms differ by the initial treatment received and by subsequent probabilities, but are conceptually the same.

A Markov model (or state-transition model) was constructed to calculate the incremental costs and benefits associated with SABR and the SoC over a five-year time horizon. A Markov model framework was chosen as it reflects both the recurring health states over a long period of time and the data that were available. This approach is characterized by hypothetical patients transitioning between mutually exclusive health states. The identified health states are based on discussions with clinical experts and a review of the literature. Hypothetical patients transition between health states at specified time points (model cycles) based on assigned probabilities. Importantly, conventional Markov models use static or fixed transition probabilities for the entire model time horizon whereas our model relaxes this assumption and incorporates time-dependent transition probabilities, based on the SABR-COMET trial. We assumed a model cycle length of three months. The conceptual model for each of the two treatment arms is presented in Figure 1. 

In the SABR treatment arm (Figure 1), all patients receive one treatment course of SABR for the initial model cycle before transitioning into the ‘Stable Disease’ health state. Patients who experienced progression events, major toxicity, or treatment related complications then transitioned into the ‘Progression’ health state. Within each health state, additional events were possible. These events reflected clinical events that, for example, might occur while a patient is receiving SABR or systemic therapy. These within-cycle events determine in which health state the patients would transition to at the end of that cycle. These events occur on the basis of their respective probabilities and are associated with specific costs and utility values. All costs and utilities are discounted at a rate of 1.5% per year [9]. Costs were adjusted to 2018 Canadian dollars [10,11].

### 2.3. Model Data

This analysis was motivated by the phase II SABR-COMET trial [7]. Where possible, we used data published from SABR-COMET to inform the SABR treatment arm of our model. The structure and data for the comparator treatment arm was further shaped through discussions with experts and from the literature. Health states and their respective utility weights and costs reflect what may be experienced by patients suffering from a variety of cancers. Systemic therapy was chosen, after expert clinician input, as the most appropriate comparator as the SoC since it reflects the most realistic alternative to SABR for the patient population in BC. We have framed our model from the perspective of an institution with no existing SABR equipment, infrastructure, or personnel; therefore, patients in the SABR arm experience an upfront cost associated with the initial capital costs of the equipment (Table S1). Several health states in the SABR treatment arm included events associated with treatment-related toxicity. 

### 2.4. Budget Impact Analysis

We conducted a budget impact analysis (BIA) from the perspective of a hospital that had no existing SABR equipment/infrastructure. That is, all capital costs and human resource costs that could possibly be associated with SABR were attributed to adoption of the technology. As an example, the linear accelerator that is used to deliver SABR may be used in other therapies or services. In this example, for the purposes of the BIA, the entirety of the cost of the linear accelerator is was included in the analysis. As such, the BIA is a conservative estimate of the budgetary impact of the adoption of SABR. 

### 2.5. Probabilistic Analysis 

Time-dependent transition probabilities for the SABR arm of the model were extracted from published survival curves from the SABR-COMET trial [7]. The survival curves were projected forward an additional five years using Weibull parametric survival regression [12] (Figures S3 and S4). Transition probabilities were made probabilistic for observed time periods using the statistical analysis software *R* and package FlexSurv [13] to calculate variance from the Hessian at the maximum, transformed back to the original scale of the parameters at each time point. Future transition probabilities were made probabilistic using the standard error of prediction at each time point [14]. Time-dependent transition probabilities for the comparator arm of the model were taken from the literature (Table 1) [7,15]. For the ten-year time horizon analysis, projected transition probabilities based on overall survival and PFS were assumed to be equal for the SABR arm and standard of care arm of the model after five years. That is, given the data available, we assumed a non-durable effect of SABR beyond the time observed in the trial. For each model parameter, a distribution representing second order uncertainty was assigned and sampled in the probabilistic analysis. We computed 10,000 iterations of the model for both a five-year (reference case analysis) and a ten-year time horizon (as a sensitivity analysis). To make the model probabilistic, costs were selected from a gamma distribution; utility weights and transition probabilities were sampled from a beta distribution. If unavailable, the variance of cost parameters was assumed to be 25% and 10% for utility and probability values. 

### 2.6. Model Outcomes

Costs, life years gained (LYG), and quality-adjusted life-years (QALYs) were calculated for each therapy to assess the impact therapies have on quality of life, survival, and the efficient use of health care resources. The main outcome of the probabilistic analysis was the incremental cost-effectiveness ratio (ICER). 

## 3. Results

**Figure 2:**
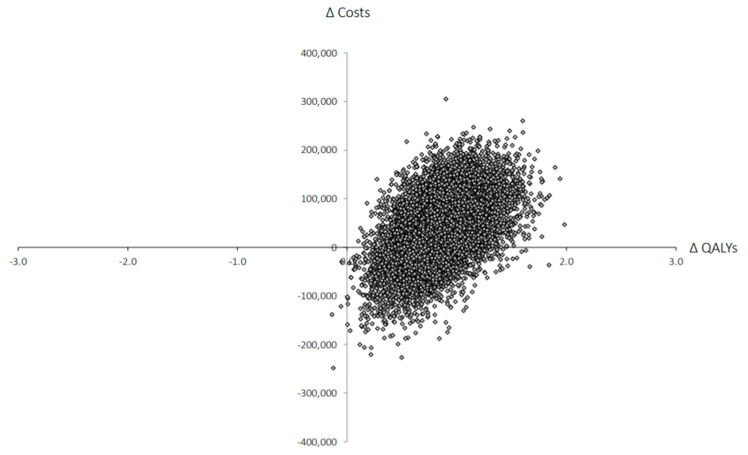
Incremental cost-effectiveness ratio (ICER) scatter plot presenting results of the five-year time horizon, based on 10,000 iterations of the probabilistic analysis. (QALYs = quality-adjusted life-years).

**Figure 3:**
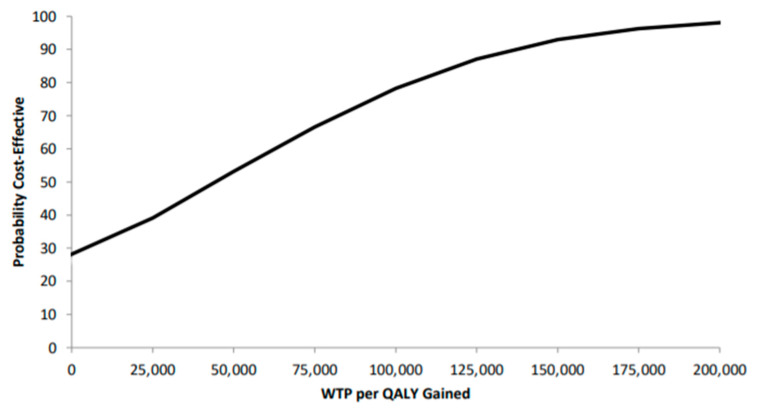
Cost-effectiveness acceptability curve (CEAC) of the ICER using a five-year model time horizon. QALY: quality-adjusted life-year; WTP: willingness-to-pay.

The results of our analysis suggest that treatment with SABR for patients with oligometastatic cancer was more costly than the systemic therapy (SoC) regimen (CAD 495,853 (95% CI: CAD 410,330–577,745) compared to CAD 457,365 (95% CI: CAD 385,331–536,483), respectively), but also generated more QALYs (1.88 (95% CI: 1.44–2.35) versus 1.03 (95% CI: 0.88–1.21), respectively) using a five-year time horizon. This resulted in an ICER of CAD 45,279 per QALY gained (Table 2). Similarly, SABR generated 0.61 (95% CI: (−0.16)–(1.34)) additional life-years compared to the standard care which resulted in an incremental cost per LYG of CAD 63,095. Results from our probabilistic analysis suggest that SABR appears cost-effective in 78% of simulations at the commonly cited threshold of CAD 100,000 per QALY gained (Figure 2 and Figure 3). 

In the analysis using the ten-year time horizon, SABR was more costly than the SoC, largely due to patients receiving additional rounds of systemic therapy post-SABR treatment (incremental cost: CAD 291,544 (95% CI: CAD 28,937–613,465) and a positive incremental gain in QALYs (1.21 (95% CI: 0.60–1.81) and resulting ICER of CAD 240,945 per QALY (Table 3). The increase in the ICER relative to reference case analysis stems from the uncertainty associated with the maturity of the evidence for SABR and specifically the durability of the treatment effect of SABR over a longer time horizon (see Figures S1 and S2). 

The results of our budget impact analysis suggest that, for centers with no SABR-related technology or infrastructure, the budget impact in the first year is estimated to be substantial, and largely driven by upfront capital costs (CAD 5,301,805). The associated requirement for human resources, machinery, and software are offset to a degree by the high cost of systemic therapies for treatment of oligometastatic cancers. By year five, given the high costs of systemic therapy and no further requirement for capital equipment, SABR results in a net savings to the health system (−CAD 11,277,333)**.**

## 4. Discussion

This analysis demonstrates that SABR is a potentially cost-effective treatment option relative to systemic therapy in patients with oligometastatic disease. In our reference case analysis, SABR was associated with an increase in both LYG and QALYs, using a five-year model time horizon. While associated with considerable capital and human resource costs, based on our probabilistic analysis, SABR appears to be cost-effective at the commonly cited threshold of CAD 100,000 per QALY gained. Importantly, given the uncertainty associated with the evidence for SABR, these results are particularly sensitive to the model time horizon and assumptions made about the durability of the treatment effect from SABR therapy. Therefore, whether or not SABR technology is adopted and implemented will depend on decision-makers tolerance for uncertainty. 

The results of our analysis should be considered cautiously. While our approach used appropriate and robust modeling techniques consistent with Canadian guidelines for the conduct of economic evaluation, it must be acknowledged that the estimates of the effectiveness of SABR result from a phase II clinical trial. As such, the real-world effectiveness of SABR remains yet to be fully understood and could benefit from a subsequent phase III trial to reduce uncertainty; this trial is currently underway [27]. Importantly, various aspects of SABR-COMET could be enhanced to aid decision-making, such as specification of a comparator that would more accurately reflect treatment decisions, additional sample size, and a longer follow-up period. However, these limitations are characteristic of phase II trials generally, and are not specifically criticisms of SABR-COMET.

Economic evaluation in the context of medical devices such as SABR poses additional analytical challenges largely stemming from the iterative nature of device development, high up-front capital costs, and the methods of evidence generation [28]. For example, to provide SABR to patients in a particular center, there is a requirement for a specialized linear accelerator, space within the treatment center, software licenses, stabilization equipment, as well as human resources to deliver this therapy. Accurate data on the costs of each of these components are hard to attain and attribute to SABR as the component costs are not always solely associated with SABR, or for SABR for treatment of oligometastatic cancer alone. Investment in these technologies differs from that of medications, particularly given the uncertainty associated with the effectiveness of SABR. At a minimum, implementation of SABR should come with continued data collection on patients undergoing the therapy. This will be especially important given that the indications for SABR may expand in the future based on the number of trials currently underway worldwide. 

Previously conducted cost-effectiveness analyses have found results consistent with our own [19,29,30]. A Canadian study compared SABR to surgery, supportive care, and other forms of radiotherapy for inoperable and borderline inoperable NSCLC patients [19]. The results suggested that SABR may be associated with lower incremental costs and higher incremental benefits than its comparators. The authors concluded that an increased use of SABR would result in significant cost-savings and survival gains [19]. More recently, Qu et al. [30] also using SABR-COMET data reported an ICER of CAD 37,250 per QALY gained, under more favorable assumptions than our analysis. For example, in our analysis, we have assumed that the durability of the treatment effect from SABR does not extend beyond the observed clinical trial period, given the uncertainty of the evidence acquired from the phase II clinical trial.

The estimated ICER in our analysis was unsurprisingly driven both by the substantial upfront costs of implementing SABR and the small incremental HRQoL benefit from treatment with SABR. The results of our cost-utility analysis estimating incremental QALYs between the two treatment arms aligns with findings from SABR-COMET that there were no appreciable improvements in HRQoL. The trial authors reported no significant differences as measured using the FACT-G instrument at six months (82.5 in the control group versus 82.6 in SABR group, *p* = 0.99). The authors also reported no statistically significant differences in specific domains of the FACT-G between treatment arms nor in any of the domains [7].

Implementing new health technologies, particularly those requiring additional infrastructure or large capital investments (such as SABR) on the basis of early phase trials should be undertaken cautiously. From a budget impact perspective, these investments can impose significant burdens on payers’ budgets but differ from conventional drugs where the cost comes from procurement of the drug for an individual patient (i.e., additional cost per patient), instead requiring a considerable investment and restructuring at the time of implementation. Similarly, disinvestment or abandonment of SABR would be considerably more difficult than for drugs should further confirmatory trial evidence not support the findings of the initial trial. Given the overlap in the potential uses of this equipment, the investment in SABR for OM disease can be transitioned to SABR for treatment of primary cancers of various disease sites (i.e., liver, lung kidney, and prostate cancer).

### Limitations

There are several limitations to this analysis which must be acknowledged. First, individual-level survival data from the SABR-COMET phase II trial were not available which meant a requirement for a number of simplifying assumptions in our analyses. The time-dependent transition probabilities used for the SABR treatment arm of this model were estimated using a digitized extraction procedure from survival curves published in SABR-COMET [7]. We extrapolated survival curves from the SABR-COMET trial over a ten-year time horizon. The estimated uncertainty of survival time after five years increases over time, and any projection of data forward into the future, while necessary and conducted appropriately, introduces additional uncertainty. Importantly, SABR can be used as treatment for a variety of types of cancer; SABR-COMET investigated predominantly breast, colorectal, lung, and prostate cancers. Given the variety of systemic treatment options for these cancers, a comparison with a treatment arm that is constant across cancer types is potentially problematic. Moreover, the management of cancers in this analysis is evolving rapidly which will have significant implications for both costs and outcomes. This assumption, however, represents a very conservative approximation of a real-world comparison given the relatively low drug costs included in the analysis relative to future drug costs and availability in some jurisdictions. We were required to make a number of assumptions around the costs of SABR, including additional events in that could occur during or post-treatment. Given that SABR is a relatively new technology, values for model parameters were taken from the literature; our choice of parameters in these instances were conservative estimates of implementing a new technology such as SABR. An update from the SABR-COMET trial was recently published [31] which reported results over an extended time period. These results, however, were not incorporated into our analysis as the number of patients included in this extended follow-up period was prohibitively low. Finally, our analysis does not include equity or environmental considerations associated with adoption of SABR, and focuses solely on the adoption of SABR from the perspective of a provincial cancer agency.

In conclusion, for patients with oligometastatic cancer, our analysis suggests that SABR is associated with higher costs, life-years, and QALYs. The resulting ICER suggests that SABR may be cost-effective at a commonly cited threshold of CAD 100,000 per QALY gained. Given that the data used to populate our economic model come from a phase II clinical trial, our results are highly sensitive to the model time horizon, and the durability of the treatment effect from SABR. Therefore, given this uncertainty and large upfront cost of SABR, the importance of further clinical and health economic research is warranted. 

## Figures and Tables

**Table 1:** Model parameters used for the SABR and SoC arms.

Health States	Value	Distribution	Source
*Costs*			
SABR Time-Zero Costs	$89,696.22	Gamma	BC Cancer internal estimate
SABR No Toxicity	$3563.38	Gamma	BC Cancer internal estimate
SABR Minor Toxicity	$3646.89	Gamma	Shah et al. (2013) [16]
SABR Major Toxicity	$4800.10	Gamma	Minion et al. (2015) [17]
Stable Disease No Toxicity	$12,737.47	Gamma	Fox et al. (2008) [18]
Stable Disease Minor Toxicity	$12,820.98	Gamma	Fox et al. (2008) [18]
Stable Disease Major Toxicity	$14,063.44	Gamma	Shah et al. (2013) [16]
Progression	$43,397.98	Gamma	Calculated
Systemic Therapy	$43,397.98	Gamma	Calculated
Stable Disease	$12,820.97	Gamma	Fox et al. (2008) [18]
Supportive Care	$3959.91	Gamma	Louie et al. (2014) [19]
*Utilities*			
SABR No Toxicity	0.73	Beta	Zemplenyi et al. (2019) [20]; Stewart et al. (2005) [21]
SABR Minor Toxicity	0.60	Beta	Zemplenyi et al. (2019) [20]; Stewart et al. (2005) [21]
SABR Major Toxicity	0.46	Beta	Grutters et al. (2019) [22]
Stable Disease No Toxicity	0.78	Beta	Zemplenyi et al. (2019) [20] ; Stewart et al. (2005) [21]
Stable Disease Minor Toxicity	0.73	Beta	Wen et al. (2014) [23]
Stable Disease Major Toxicity	0.59	Beta	Wen et al. (2014) [23]
Progression	0.62	Beta	Lester-Coll et al. (2016) [24]
Stable Disease	0.78	Beta	Zemplenyi et al. (2019) [20]
Systemic Therapy	0.62	Beta	Lester-Coll et al. (2016) [24] ; Doyle et al. (2008) [25]
Supportive Care	0.29	Beta	de Kok et al. (2009) [26]

**Table 2:** Results from the reference case analysis assuming a five-year time horizon. Average estimates from the probabilistic analysis with 95% confidence intervals presented in brackets.

	Costs	QALYs	Incremental Costs	Incremental QALYs	ICER
SABR	$495,853 (410,330–577,745)	1.88 (1.44–2.35)	38,488	0.85	$45,279
SoC	$457,365 (385,331–536,483)	1.03 (0.88–1.21)			

**Table 3:** Sensitivity analysis assuming a ten-year time horizon. 95% confidence intervals are presented in brackets.

	Costs	QALYs	Incremental Costs	Incremental QALYs	ICER
SABR	$756,622 (507,713–1,069,635)	2.26 (1.94–2.65)	$291,544	1.21	$240,945
SoC	$465,078 (389,160–559,224)	1.05 (0.88–1.21)			

## Data Availability

The data presented in this study are available in this article, in the cited literature, and Supplementary Materials.

## Supplementary Materials

The following are available online at https://www.mdpi.com/article/10.3390/curroncol28030172/s1, Figure S1. Using a ten year time horizon, scatter plot of probabilistic incremental cost-effectiveness ratios (ICERs), Figure S2. Cost-effectiveness acceptability curve (CEAC) of the ICER using a ten year time horizon, Table S1. Estimated upfront time-zero cost estimate for the introduction of SABR based internal BC Cancer costing, Table S2. Cancer costs by site, Table S3. Patient distribution for the SABR arm in SABR-COMET, Table S4. Patient distribution for the SABR arm in SABR-COMET normalized to exclude ‘Other’, Table S5. Patient distribution for the SABR arm in SABR-COMET weighted cost (per 3 month period), Figure S3. Extrapolated overall survival curve for the SABR model arm, Figure S4. Extrapolated progression-free survival curve for the SABR model arm.

## References

[1] Murray P., Franks K., Hanna G.G. (2017). A systematic review of outcomes following stereotactic ablative radiotherapy in the treatment of early-stage primary lung cancer. Br. J. Radiol..

[2] Simeonova A.O., Fleckenstein K., Wertz H., Frauenfeld A., Boda-Heggemann J., Lohr F., Wenz F. (2012). Are three doses of stereotactic ablative radiotherapy (SABR) more effective than 30 doses of conventional radiotherapy?. Transl. Lung Cancer Res..

[3] Potters L., Kavanagh B., Galvin J.M., Hevezi J.M., Janjan N.A., Larson D.A., Mehta M.P., Ryu S., Steinberg M., Timmerman R. (2010). American society for therapeutic radiology and oncology (ASTRO) and American college of radiology (ACR) practice guideline for the performance of stereotactic body radiation therapy. Int. J. Radiat. Oncol. Biol. Phys..

[4] Choy H. (2017). SABR as First Line Treatment Option. J. Thorac. Oncol..

[5] Olson R., Liu M., Bergman A., Lam S., Hsu F., Mou B., Berrang T., Mestrovic A., Chng N., Hyde D. (2018). Population-based phase II trial of stereotactic ablative radiotherapy (SABR) for up to 5 oligometastases: SABR-5. BMC Cancer.

[6] Xhaferllari I., El-Sherif O., Gaede S. (2016). Comprehensive dosimetric planning comparison for early-stage, non-small cell lung cancer with SABR: Fixed-beam IMRT versus VMAT versus TomoTherapy. J. Appl. Clin. Med. Phys..

[7] Palma D.A., Olson R., Harrow S., Gaede S., Louie A.V., Haasbeek C., Mulroy L., Lock M., Rodrigues G.B., Yaremko B.P. (2019). Stereotactic ablative radiotherapy versus standard of care palliative treatment in patients with oligometastatic cancers (SABR-COMET): A randomised, phase 2, open-label trial. Lancet.

[8] Senan S., Olson R., Harrow S., Gaede S., Louie A., Haasbeek C., Mulroy L., Lock M., Rodrigues G., Yaremko B. (2018). Stereotactic ablative radiotherapy for oligometastatic cancers: Efficacy and toxicity results from the randomized SABR-COMET Trial. Ann. Oncol..

[9] Canadian Agency for Drugs and Technologies in Health (2017). Guidelines for the Economic Evaluation of Health Technologies in Canada.

[10] Bank of Canada Bank of Canada Inflation Calculator. https://www.bankofcanada.ca/rates/related/inflation-calculator/.

[11] USD Historical Exchange Rates (US Dollar)—X-Rates. https://www.x-rates.com/historical/?from=USD&amount=1&date=2014-07-29.

[12] Newcombe P.J., Raza Ali H., Blows F.M., Provenzano E., Pharoah P.D., Caldas C., Richardson S. (2017). Weibull regression with Bayesian variable selection to identify prognostic tumour markers of breast cancer survival. Stat. Methods Med. Res..

[13] Jackson C. (2016). Flexsurv: A platform for parametric survival modeling in R. J. Stat. Softw..

[14] Mendenhall W., Sincich T. (2012). A Second Course in Statistics: Regression Analysis.

[15] Guest J.F., Ruiz F.J., Greener M.J., Trotman I.F. (2006). Palliative care treatment patterns and associated costs of healthcare resource use for specific advanced cancer patients in the UK. Eur. J. Cancer Care.

[16] Shah A., Hahn S.M., Stetson R.L., Friedberg J.S., Pechet T.T.V., Sher D.J. (2013). Cost-effectiveness of stereotactic body radiation therapy versus surgical resection for stage I non-small cell lung cancer. Cancer.

[17] Minion L.E., Bai J., Monk B.J., Robin Keller L., Ramez E.N., Forde G.K., Chan J.K., Tewari K.S. (2015). A Markov model to evaluate cost-effectiveness of antiangiogenesis therapy using bevacizumab in advanced cervical cancer. Gynecol. Oncol..

[18] Fox K.M., Brooks J.M., Kim J. (2008). Metastatic non–small cell lung cancer: Costs associated with disease progression. Am. J. Manag. Care.

[19] Louie A.V., Rodrigues G.B., Palma D.A., Senan S. (2014). Measuring the population impact of introducing stereotactic ablative radiotherapy for stage i non-small cell lung cancer in canada. Oncologist.

[20] Zemplényi A.T., Kaló Z., Kovács G., Farkas R., Beöthe T., Bányai D., Sebestyén Z., Endrei D., Boncz I., Mangel L. (2018). Cost-effectiveness analysis of intensity-modulated radiation therapy with normal and hypofractionated schemes for the treatment of localised prostate cancer. Eur. J. Cancer Care.

[21] Stewart S.T., Lenert L., Bhatnagar V., Kaplan R.M. (2005). Utilities for prostate cancer health states in men aged 60 and older. Med. Care.

[22] Grutters J.P.C., Pijls-Johannesma M., Ruysscher D.D., Peeters A., Reimoser S., Severens J.L., Lambin P., Joore M.A. (2010). The cost-effectiveness of particle therapy in non-small cell lung cancer: Exploring decision uncertainty and areas for future research. Cancer Treat. Rev..

[23] Wen F., Yao K., Du Z.-D., He X.-F., Zhang P.-F., Tang R.-L., Li Q. (2014). Cost-effectiveness analysis of colon cancer treatments from MOSIAC and No. 16968 trials. World J. Gastroenterol. WJG.

[24] Lester-Coll N.H., Rutter C.E., Bledsoe T.J., Goldberg S.B., Decker R.H., Yu J.B. (2016). Cost-effectiveness of surgery, stereotactic body radiation therapy, and systemic therapy for pulmonary oligometastases. Int. J. Radiat. Oncol..

[25] Doyle S., Lloyd A., Walker M. (2008). Health state utility scores in advanced non-small cell lung cancer. Lung Cancer Amst. Neth..

[26] de Kok I.M.C.M., van Ballegooijen M., Habbema J.D.F. (2009). Cost-effectiveness analysis of human papillomavirus vaccination in the netherlands. JNCI J. Natl. Cancer Inst..

[27] Olson R., Mathews L., Liu M., Schellenberg D., Mou B., Berrang T., Harrow S., Correa R.J.M., Bhat V., Pai H. (2020). Stereotactic ablative radiotherapy for the comprehensive treatment of 1–3 Oligometastatic tumors (SABR-COMET-3): Study protocol for a randomized phase III trial. BMC Cancer.

[28] Tarricone R., Torbica A., Drummond M. (2017). Challenges in the assessment of medical devices: The medtecHTA project. Health Econ..

[29] Smith B.D., Jiang J., Chang J.Y., Welsh J., Likhacheva A., Buchholz T.A., Swisher S.G., Shirvani S.M. (2015). Cost-effectiveness of stereotactic radiation, sublobar resection, and lobectomy for early non-small cell lung cancers in older adults. J. Geriatr. Oncol..

[30] Qu M.X., Chen Y., Zaric G., Senan S., Olson R.A., Harrow S., John-Baptiste A., Gaede S., Mulroy L., Schellenberg D. (2019). Is SABR cost-effective in oligometastatic cancer? An economic analysis of SABR-comet. Int. J. Radiat. Oncol. Biol. Phys..

[31] Palma D.A., Olson R., Harrow S., Gaede S., Louie A.V., Haasbeek C., Mulroy L., Lock M., Rodrigues G.B., Yaremko B.P. (2020). Stereotactic ablative radiotherapy for the comprehensive treatment of oligometastatic cancers: Long-term results of the SABR-COMET phase II randomized trial. J. Clin. Oncol..

