# Supplementary material for: Cost-Effectiveness Analysis of Stereotactic Ablative Body Radiotherapy for the Treatment of Oligometastatic Tumors versus Standard of Care
Source: Curr Oncol. 2021 May 13;28(3):1857–66. doi: 10.3390/curroncol28030172 (PMC8161824; doi:10.3390/curroncol28030172)
Supplement: Supplementary file 1 [file curroncol-28-00172-s001.zip › curroncol-1059921-supplementary.pdf]

SUPPLEMENTARY MATERIAL

Cost-Effectiveness Analysis of Stereotactic Ablative Body Radiotherapy for the Treatment of Oligometastatic Tumors Versus Standard of Care

Adam J.N. Raymakers, David Cameron, Scott Tyldesley and Dean A. Regier

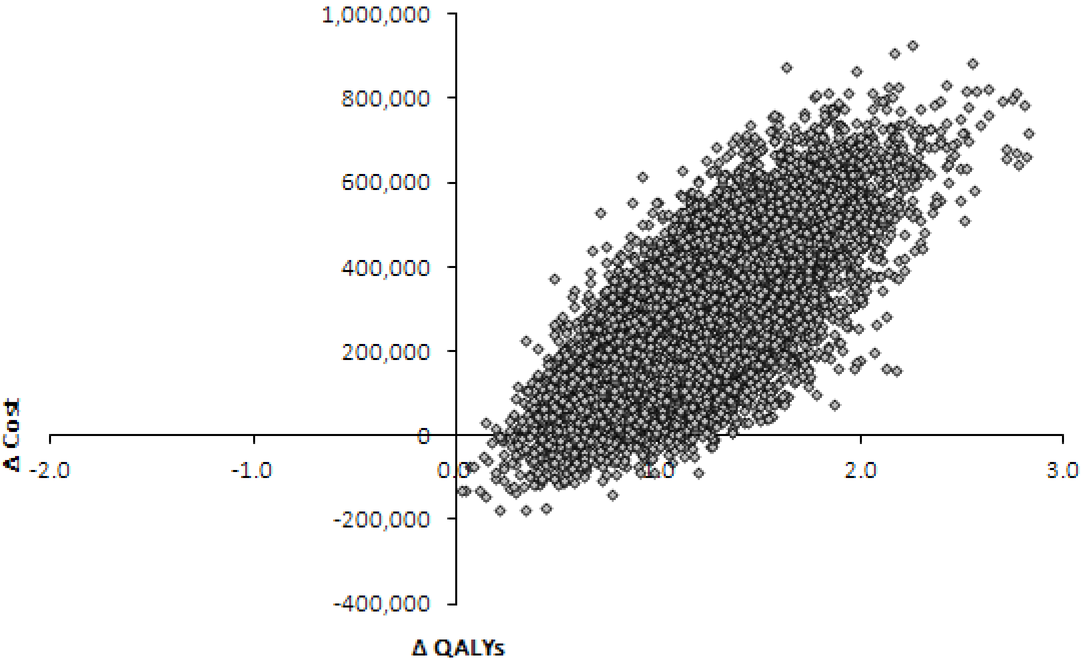

Figure S1. Using a ten year time horizon, scatter plot of probabilistic incremental cost-effectiveness ratios (ICERs).

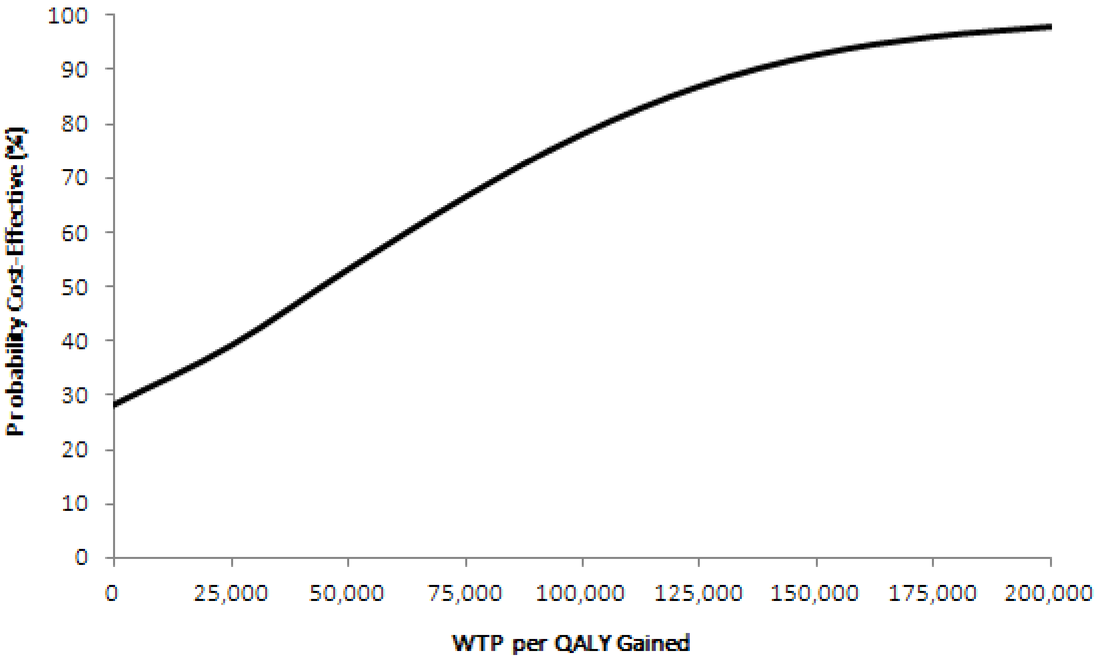

Figure S2. Cost-effectiveness acceptability curve (CEAC) of the ICER using a ten year time horizon. QALY: quality-adjusted life-year; WTP: willingness-to-pay. .

Table S1. Estimated upfront time-zero cost estimate for the introduction of SABR based internal BC Cancer costing.

**Equipment & Training Costs**	Cost (CAD$)
Immobilization device	$61,959
Linear accelerator	$4,135,000
Additional hardware and software	$1,236,269
Software training costs	$3,000
Dedicated planning workstation	$79,288
Auto-segmentation and auto-contouring software	$500,000
Tracking devices	$350,000
Training (including estimated travel for 1 RT & 1 physicist)	$2915
**Total:**	$6,368,431

An average of 71 SBRT treatments were conducted at each cancer treatment site in BC in 2017 (the most recent observed year that data is available for).Equipment & Training Costs (upfront costs) per patient: $6,368,431/71 = **$89,696** (2018 CAD$).

Costs have been adjusted to 2018 CAD$ using a historical currency conversion tables and adjusted for inflation.

Table S2. Cancer costs by site.

Breast

Capecitabine (BRAVCAP) was used for the treatment of metastatic breast cancer. Dosage was assumed to be 2000 to 2500 mg/ day for two of every three weeks, typically 6-8 cycles. Cost was assumed to be $6.97 per day (2015 CAD$)

$6.97 * 90 = $627.30 (2015 CAD$)

3 month cost: **$659.87** (2018 CAD$)

GI

Gemcitabine + Cisplatin (GIAVPG) used for the treatment of metastatic colon cancer. Dosage was assumed to be gemcitabine 1000 mg/m2 on days 1 and 8 of 21 day cycle and cisplatin 25 mg/m2 on days 1 and 8 of 21 day cycle. Monthly drug cost was assumed to be $44,574 (2016 USD$).

44,574 * 3 = 133,722 (2016 USD$)

3 month cost: **$186,004.39 (2018 CAD$)**

Lung

Erlotinib (LUAVERL) was used for the treatment of metastatic lung cancer. Dosage was assumed to be 150mg taken daily.

Cost was assumed to be $5,751 (2015 USD$$) per month (150mg taken daily). Includes the administration cost of one outpatient office visit for initial prescription. All drug prices are average wholesale price minus 17%).

5,751*3 = $17,253 (2015 USD$)

3 month cost: **$22,601.43** (2018 CAD$)

Prostate

Standard of care in castration-resistant prostate cancer was assumed to be docetaxel and prednisone.

Dosage was assumed to be Prednisone 5 mg twice daily and Docetaxel 75 mg/m^2 administered every 3 weeks.

Cost was assumed to be £454.53 per 80 mg vial (2015) for docetaxel and

Prednisone oral solution (5 mg/mL) was assumed to be $119 for a supply of 30 ml

6 month cost of docetaxel: 8.666*454.53 = 3,930.29 (2015 £)

= **$8,351.45** (2018 CAD$)

6 month cost of Prednisone: 60*$119 = 7,140 (2019 USD$)

= **$9,181.71** (2018 CAD$)

Total 6 month cost: $17,533.16 (2018 CAD$)

Total 3 month cost: **$8,766.58** (2018 CAD$)

Table S3. Patient distribution for the SABR arm in SABR-COMET.

Site	Percentage
Breast	20%
Colorectal	14%
Lung	18%
Prostate	21%
Other	26%

Table S4. Patient distribution for the SABR arm in SABR-COMET normalized to exclude 'Other'.

Site	Percentage
Breast	27%
Colorectal	19%
Lung	24%
Prostate	28%

Table S5. Patient distribution for the SABR arm in SABR-COMET weighted cost (per 3 month period):.

Site	Cost (2018 CAD)
Breast	$178.16
Colorectal	$35,340.84
Lung	$5,424.34
Prostate	$2,454.64
**Total:**	**$43,397.98**

In the Progression/Systemic Therapy health state, patients will experience costs of **$43,397.98** per 3 month period. Extrapolated overall survival and progression-free survival curves from the SABR-COMET trial – 10 year time horizon**.**

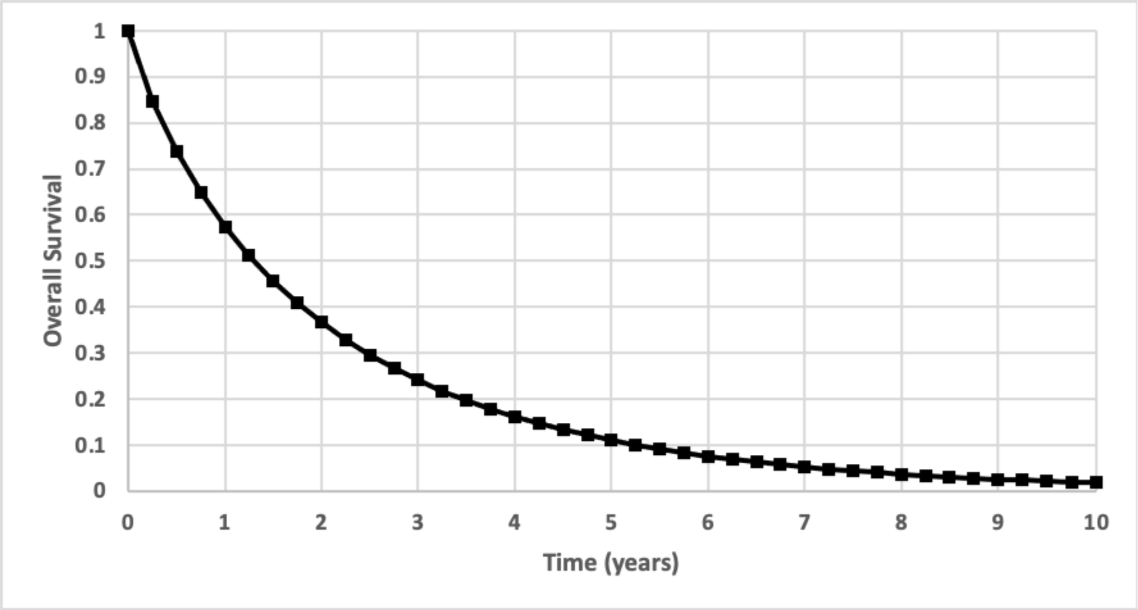

Figure S3. Extrapolated overall survival curve for the SABR model arm.

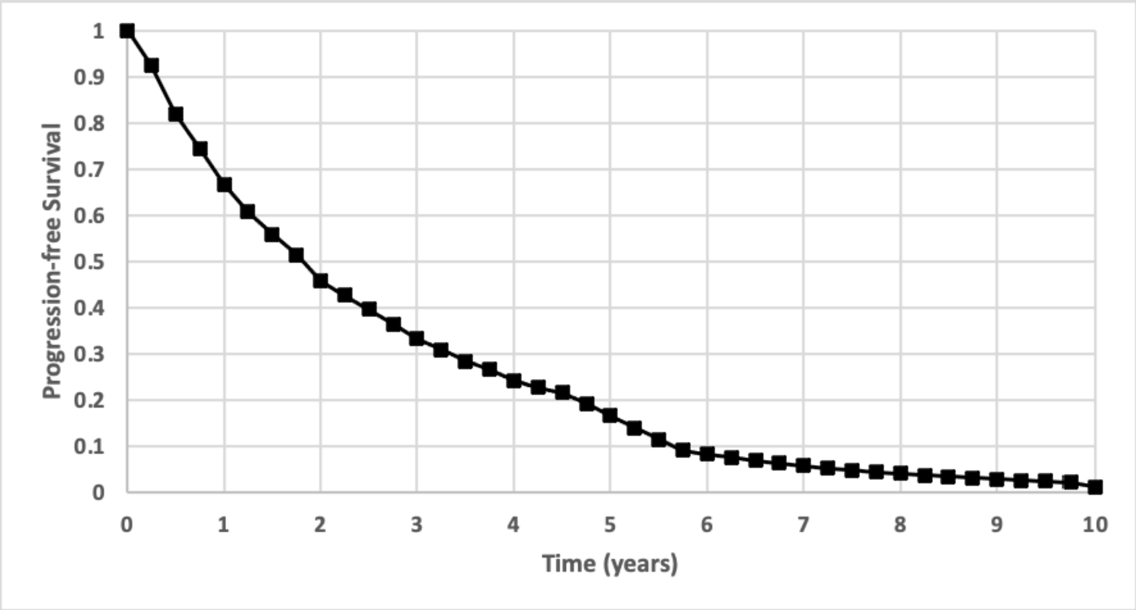

Figure S4. Extrapolated progression-free survival curve for the SABR model arm.
